# Compulsive avoidance in youths and adults with OCD: an aversive pavlovian-to-instrumental transfer study

**DOI:** 10.1038/s41398-024-03028-1

**Published:** 2024-07-26

**Authors:** Aleya A. Marzuki, Paula Banca, Sara Garofalo, Luigi A. E. Degni, Daniela Dalbagno, Marco Badioli, Akeem Sule, Muzaffer Kaser, Anna Conway-Morris, Barbara J. Sahakian, Trevor W. Robbins

**Affiliations:** 1 Behavioural and Clinical Neuroscience Institute, Department of Psychology, University of Cambridge, Cambridge, UK; 2 Department of Psychology, School of Medical and Life Sciences, Sunway University, Petaling Jaya, Selangor Malaysia; 3 Department of Psychology, University of Bologna, Bologna, Italy; 4 Department of Psychiatry, School of Clinical Medicine, University of Cambridge, Cambridge, UK; 5 Cambridgeshire and Peterborough NHS Foundation Trust, Cambridge, UK

**Keywords:** Addiction, Human behaviour

## Abstract

Compulsive behaviour may often be triggered by Pavlovian cues. Assessing how Pavlovian cues drive instrumental behaviour in obsessive-compulsive disorder (OCD) is therefore crucial to understand how compulsions develop and are maintained. An aversive Pavlovian-to-Instrumental transfer (PIT) paradigm, particularly one involving avoidance/cancellation of negative outcomes, can enable such investigation and has not previously been studied in clinical-OCD. Forty-one participants diagnosed with OCD (21 adults; 20 youths) and 44 controls (21 adults; 23 youths) completed an aversive PIT task. Participants had to prevent the delivery of unpleasant noises by moving a joystick in the correct direction. They could infer these correct responses by learning appropriate response-outcome (instrumental) and stimulus-outcome (Pavlovian) associations. We then assessed whether Pavlovian cues elicited specific instrumental avoidance responses (specific PIT) and induced general instrumental avoidance (general PIT). We investigated whether task learning and confidence indices influenced PIT strength differentially between groups. There was no overall group difference in PIT performance, although youths with OCD showed weaker specific PIT than youth controls. However, urge to avoid unpleasant noises and preference for safe over unsafe stimuli influenced specific and general PIT respectively in OCD, while PIT in controls was more influenced by confidence in instrumental and Pavlovian learning. Thus, in OCD, implicit motivational factors, but not learnt knowledge, may contribute to the successful integration of aversive Pavlovian and instrumental cues. This implies that compulsive avoidance may be driven by these automatic processes. Youths with OCD show deficits in specific PIT, suggesting cue integration impairments are only apparent in adolescence. These findings may be clinically relevant as they emphasise the importance of targeting such implicit motivational processes when treating OCD.

## Introduction

Conditioned cues may act as triggers for compulsions in obsessive-compulsive disorder (OCD) and further act as motivational influences that increase their intensity [1]. However, the role of conditioned associations between environmental cues and aversive events in influencing this avoidance behaviour is under-researched in clinical-OCD. Pavlovian-to-Instrumental Transfer (PIT) provides a paradigm for understanding how conditioned cues influence learnt instrumental actions to seek rewards or avoid punishment [2].

Typically, PIT involves 3 key stages: 1) an instrumental phase - establishing a contingent relationship between distinct instrumental actions and rewarding/punishing outcomes (*Experimental Example:* learning that moving a joystick left leads to a gain in points; *Real-life Example:* learning that handwashing alleviates distress caused by possible contamination), 2) a Pavlovian phase - implicitly learning the association between Pavlovian cues and the rewarding/punishing outcomes (*Experimental Example:* a blue seashell image predicting a gain in points; *Real-Life Example:* watching or hearing reports of a disease outbreak on television/radio, resulting in distress); and 3) the PIT phase - which probes the impact of the Pavlovian conditioned cue on eliciting or increasing the instrumental behaviour (*Experimental Example:* increased vigour in moving the joystick when encountering the blue seashell from the Pavlovian phase; *Real-life Example:* increased handwashing when hearing or watching news related to the disease outbreak). PIT comprises 2 distinct processes: specific and general. Specific PIT refers to the selective effect of a Pavlovian cue on responses related to the same outcome (*Experimental Example*: moving left more often when seeing the same blue seashell, as this was the side that was reinforced; *Real-life Example:* washing one’s hands in response to specific reports of the disease outbreak) while general PIT probes the overall motivational influence of a similar Pavlovian cue on instrumental approach/avoidance behaviour (*Experimental Example:* increased joystick movement towards a red seashell; *Real-life Example:* increased distress and urge to handwash following any emotionally-charged report, e.g., a traffic accident).

PIT tasks are useful for studying psychopathology as they can probe how goal-directed outcome-response associations established in the instrumental phase transform to become habitual stimulus-response associations in the PIT phase [3], as responses are conducted under extinction (i.e., no outcomes are delivered). For instance, they have characterised how initial goal-directed recreational alcohol or drug use shifts towards more habitual usage, triggered by Pavlovian cues in the environment (e.g., noise coming from a pub) [1, 4–6]. Additionally, it is posited that separate learning mechanisms underlie the types of transfer in the task, whereby specific PIT reflects more model-based behaviour as it involves the matching of the correct instrumental response to a conditioned stimulus in order to achieve a desired outcome, while general transfer is elicited from the implicit motivational properties of the Pavlovian cue, and is hence more reflective of model-free behaviour [7]. Concretely, specific transfer has been discovered to be associated with higher order cognitive skills but not general transfer [8].

In contrast to other psychiatric disorders, PIT research in OCD is sparse [1], but beginning to gain traction; recent work in clinical-OCD have reported specific PIT deficits in patients [9, 10] while general PIT is preserved [10]. These findings resonate with reported OCD-related dysfunctions in the goal-directed brain system [11–13]. However, these previous studies have used PIT paradigms with appetitive elements, whereas punishment avoidance may be more relevant to OCD as compulsions are often conducted to prevent harm [14] and seminal research has found goal-directed deficits in patients with OCD on aversive tasks (i.e., participants with OCD continue to exert effort to avoid previously aversive stimuli that is currently ‘safe’) [15–17]. Thus, a PIT paradigm in which participants are tasked with learning to prevent or cancel aversive outcomes may be able to trigger avoidance tendencies in OCD leading to stronger overall PIT effects, in contrast to impaired PIT seen in appetitive tasks.

Additionally, prior cognitive research indicates OCD is associated with atypical associative learning, encompassing abnormalities in how feedback and prior knowledge are integrated into decision-making [18–23]. In particular, research characterises OCD as being associated with a dissociation between confidence (meta-cognition) and action, as meta-cognitive processes do not appear to inform decision-making (which tends to be aberrant) [22, 24]. These findings may model the ego-dystonic nature of OCD, where obsessions and compulsions are disproportionate from known information about the self and external world. Past research has yet to fully investigate whether this dissociation informs avoidance behaviour in this population. This research gap may also be suitably addressed using aversive PIT methods.

The present study utilised an aversive PIT paradigm [3] to assess whether avoidance PIT is linked to compulsive behaviour. We propose two competing hypotheses: either 1) weakened specific PIT, but intact general PIT, in OCD consistent with altered goal-directed control and in line with prior work that has administered appetitive PIT tasks in OCD [9, 10] or otherwise 2) enhanced overall PIT strength driven by avoidance tendencies in this population.

We also sought to conduct exploratory analyses involving the investigation of factors influencing specific and general PIT in OCD, such as learning ability, confidence, and avoidance ratings, to ascertain specific mechanisms motivating avoidance behaviour in OCD. Based on prior cognitive work showing weakened action-confidence coupling in OCD [22], it may be that learnt knowledge and confidence would be weaker in informing PIT strength in OCD. We recruited youth and adult cases of OCD, together with age-matched controls. A cross-sectional exploratory comparison between age-groups may help to trace the ontogeny of compulsive behaviour, especially as many cases of adult OCD have initial onset of OCD symptoms in childhood/adolescence [25–27]. Moreover, few studies have directly compared adult and youth cases of OCD (and none at all in OCD PIT research) despite there being emerging evidence for divergent learning and cognition between age-subtypes [21, 28, 29].

## Methods

### Participants

This study included 41 participants diagnosed with OCD (OCD) and 44 healthy controls (CTL). The overall sample comprised two age-subgroups: adults (20 ≥ years; 21 CTL and 21 OCD) and youths (12–19 years; 23 CTL and 20 OCD). The cut-off for adulthood was determined based on guidelines by the World Health Organisation [30] and prior OCD research which has recruited similar adolescent samples [21, 24, 31]. All patients were screened by an experienced psychiatrist in an extended clinical interview supplemented by the Mini International Neuropsychiatric Interview (MINI for participants over 18, MINI-KID for participants under 18) [32, 33].

To qualify for the study, youths and adults with OCD had to meet Diagnostic and Statistical Manual of Mental Disorders-5-Text Revision diagnostic criteria for OCD, have OCD as their primary diagnosis, and score above 12 on the Children’s Yale-Brown Obsessive–Compulsive Scale 3 (for youths) and the Yale-Brown Obsessive Compulsive Scale (for adults), as a score of 12 is reported to be the optimal cut-off score for predicting remission in the CY-BOCS [34] and Y-BOCS [35]. Apart from OCD, other significant Axis I psychiatric disorders were exclusion criteria. If any participants with OCD showed elevated anxiety and depression scores (see Clinical Assessment section), a psychiatrist would evaluate whether symptoms were driven by the primary OCD diagnosis or if they were independent from it. Only those fitting the latter description were included in the study. Those with severe physical impairments affecting eyesight or motor performance were also excluded, as they were predicted to affect task performance. Control youths and adults were also screened to ensure they had no history of neurological or psychiatric illness.

Out of the 21 adults with OCD, 18 were receiving medication for their OCD: Ten adult patients were taking only selective serotonin reuptake inhibitors (SSRIs), 5 were on SSRIs alongside other medications (e.g., tricyclics or antipsychotics), and 3 were taking non-SSRI medication. Within the 20 youths with OCD, 10 were receiving SSRIs.

See Table 1 for demographic information of groups.

**Table 1:** Comparison of demographic and clinical measures in different groups.

	CTL (*N* = 44)		OCD (*N* = 41)		STATISTICS
Variable	ADULTS (*N* = 21)	YOUTHS (*N* = 23)	ADULTS (*N* = 21)	YOUTHS (*N* = 20)	
Sex (F:M)	12:9	16:7	10:11	13:7	NA
Age **	41.90 (12.05)	15.96 (1.94)	39.71 (12.69)	16.33 (1.70)	Age: Twj_1,41.58_ = 163.44, *p* < 0.0001, Group: *p* > 0.05, Group x Age: *p* > 0.05 Group and Group x Age: *p* > 0.05
Working Memory (Backwards Digit Span)^a^	7.13 (2.59)	8.52 (2.12)	7.75 (2.63)	8.10 (1.70)	Age, Group, and Group x Age: *p* > 0.05
OCI-R **^b^	5.33 (6.14)	8.52 (6.93)	31.86 (15.25)	30.35 (14.91)	Group: Twj_1, 50.71_ = 97.42, *p* < 0.0001 Age and Group x Age: *p* > 0.05
Y-BOCS/C-YBOCS Total	NA	NA	23.00 (5.09)	23.45 (5.11)	Age: *p* > 0.05
Y-BOCS/C-YBOCS Obsessions^c^	NA	NA	11.45 (2.48)	11.40 (2.37)	Age: *p* > 0.05
Y-BOCS/C-YBOCS Compulsions^c^	NA	NA	11.10 (2.65)	12.05 (3.03)	Age: *p* > 0.05
**Adult-Only Measures**					
Predicted IQ from NART^d^	117.09 (6.77)	NA	116.23 (5.77)	NA	Group: *p* > 0.05
STAI-T **^b^	31.11 (8.59)	NA	55.57 (10.77)	NA	Group: t_36_ = 7.52, *p* < 0.0001
STAI-S **^b^	27.67 (7.65)	NA	41.86 (11.64)	NA	Group: t_36_ = 4.21, *p* < 0.001
MADRS **	0.52 (1.03)	NA	8.38 (7.59)	NA	Group: t_20.74_ = 4.70, *p* < 0.001
**Youth-Only Measures**					
IQ (WASI-II)	NA	109.82 (11.67)	NA	107.25 (13.69)	Group: *p* > 0.05
BDI-Y **	NA	44.83 (6.60)	NA	59.6 (9.61)	Group: t_41_ = 5.91, *p* < 0.0001
BAI-Y **	NA	47.78 (5.43)	NA	67.00 (10.10)	Group: t_41_ = 7.91, *p* < 0.0001

Means and standard deviations [M(SD)] are reported. *NA* not applicable, *OCI-R* Obsessive- Compulsive Inventory Revised, *CY-BOCS/Y-BOCS* Child/Yale-Brown Obsessive-Compulsive Scale, *IQ* intelligence quotient, *NART* National Adult Reading Test, *STAI-T* State-Trait Anxiety Inventory – Trait, *STAI-S* State-Trait Anxiety Inventory – State, *MADRS* Montgomery-Asberg Depression Rating Scale; WASI-II, Wechsler’s Abbreviated Scale of Intelligence – II; BDI-Y, Beck Depression Inventory - Youth (t-scored); BAI - Y, Beck Anxiety Inventory - Youth (t-scored); *p < 0.05; **p < 0.01; ^a^missing data from 6 CTLs and 1 OCD (adults); ^b^missing from 3 adult CTL, ^c^missing from 1 adult OCD, ^d^missing data from 3 CTL and 1 OCD (adults)

The study involving adult participants was approved by the East of England - Cambridge South Research Ethics Committee (16/EE/0465) while the study involving adolescents was approved by the East of England - Essex Research Ethics Committee (REC 10/H030149/49). All volunteers gave written informed consent before beginning testing and were compensated at the rate of £8 per hour. Parental consent was obtained for participants under 16 years old.

### Clinical assessment

All participants completed the Obsessive-Compulsive Inventory-Revised [OCI-R [36]] to ascertain self-reported OCD symptoms. OCD symptom severity was assessed with the Yale-Brown Obsessive-Compulsive Scale (Y-BOCS [37]) in adult patients and the Children’s Yale-Brown Obsessive-Compulsive Scale (CY-BOCS [38]) in youth patients. To obtain measures of depression and anxiety respectively, adult participants were administered the Montgomery-Asberg Depression Rating Scale (MADRS [39]) and the State-Trait Anxiety Inventory (STAI) [40], while youth participants completed the Beck Depression Inventory for Youth and the Beck Anxiety Inventory for Youth [41]. Wechsler Adult Intelligence Scale IQ scores for adult patients were estimated using the National Adult Reading Test [NART, (Nelson & Willison, 1982)] as per [42]. IQ scores for youths were obtained via the Wechsler’s Abbreviated Scale of Intelligence, Second Edition [WASI-II [43]]. The Full-Scale IQ-2 subtests (FSIQ-2) from the WASI-II were used comprising the Vocabulary and Matrix Reasoning tests. Lastly, all participants completed the Digit Span Backwards test from the WASI-II as a measure of working memory performance.

### Pavlovian-to-instrumental transfer task

**Fig. 1:**
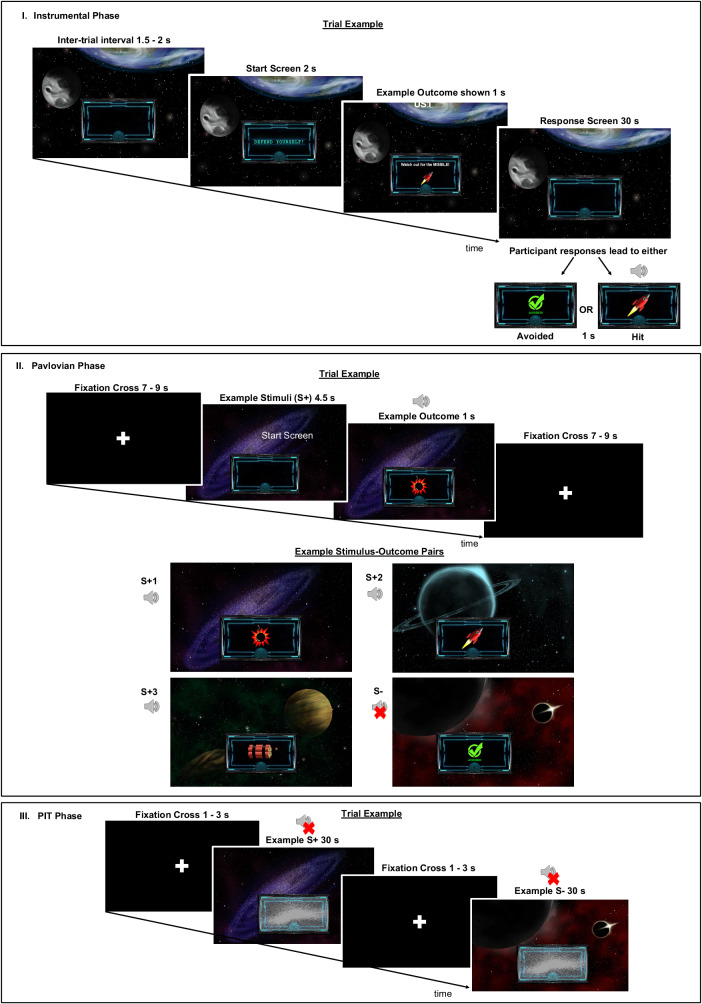
Schematic of the Pavlovian-to-Instrumental Transfer task. i. Participants learn instrumental response-outcome associations, namely between a joystick movement (left or right) and the cancellation of an aversive sound (bomb or missile). During each trial, participants are provided with a message stating, ‘Defend Yourself!’ followed by an image of either one of two unconditioned outcomes (bomb or missile). Afterwards, within 30 s, participants are required to respond either left or right to prevent ‘attacks’, which happen every 1.5–3 s. Eighty percent of the time, correct responses are followed by a message stating ‘Avoided’ while 20% of correct or all wrong responses are followed by an image of the outcome and a corresponding unpleasant noise. ii. Participants passively learn stimuli-outcome associations. Three images of galaxies (stimuli, S) are paired with aversive sounds (outcomes) while one galaxy image is ‘safe’ (no associated aversive sound). Stimulus-outcome pairing combinations are counterbalanced across participants. iii. In each trial, galaxies are shown to participants again and they must use the joystick to respond accordingly (as many times as they like within 30 s). The phase probes whether participants can integrate instrumental and Pavlovian learning when responding to the images. This phase is performed under extinction in that no noises are delivered. Key- s: second, S: Conditioned Stimuli.

The task, originally used in Garofalo & Robbins (2017), comprised 3 experimental phases (Fig. 1), namely Instrumental Conditioning, Pavlovian Conditioning, and Pavlovian-To-Instrumental Transfer (PIT). The task was presented using the software Presentation (Neurobehavioral Systems, Albany, CA, USA).

### Phase 1: instrumental conditioning

Phase 1 trains participants to create an instrumental association between goal-directed responses (moving the joystick left or right) and outcomes (two aversive noises labelled O[outcome]1 and O2). We used a ‘Space Mission’ narrative to keep participants engaged. Participants were told that they were under attack on their mission, and their goal was to avoid being hit by two possible attacks: ‘bombs’ and ‘missiles’ presented as cartoon images on screen. Each attack was associated with 1 of 2 aversive noises (O1 or O2) played at the same time as the image. Noises were delivered via a headset worn by participants. Participants were informed they could avoid hearing the noises if they moved the joystick quickly enough. They were also told that there was an optimal joystick direction that would prevent each attack. Each trial began with a visual message displaying “Defend yourself” for 2 seconds (s) followed by an image of the outcome (bomb or missile) that was about to be delivered (1 s). Each trial featured a 30 s response window, in which one of the possible outcomes would be displayed every 1.5 s to 3 s. During this time interval, participants were required to move the joystick in a certain direction (left or right) to cancel the outcomes. The association between instrumental response (left/right) and attack-type (O1/O2), counterbalanced across participants, was learnt by trial and error. If participants moved the joystick in the correct direction, the screen would display ‘Avoided’ for 1 s and no noise would be delivered. Outcomes could be avoided by moving the joystick correctly only 80% of the time, but participants were unaware of this. Instead, they were advised that if an attack still occurred despite responding in the correct direction, it was because they were not squeezing the joystick with enough strength.

During the real test phase, participants underwent 8 trials in total (4 featuring O1 and 4 featuring O2). The phase lasted approximately 8 min. At the end of this phase, we assessed explicit learning by asking participants to pair each outcome with the corresponding left or right response. Participants also rated how confident they were in their outcome-response pairings on a scale from 1–9. Lastly, they were instructed to rate how much they wanted to avoid the attacks on a scale from 1–9 (as a measure of avoidance tendencies).

Before beginning this phase, participants completed a training session with 4 blocks, without any noises delivered whenever they were ‘attacked’.

### Phase 2: pavlovian conditioning phase

This phase trains participants to learn associations between different images and outcomes. Before beginning, participants rated on a scale from 1 (most disliked) – 9 (most liked) how much 4 different outer space images appealed to them (a measure of subjective evaluation). These 4 images were used as conditioned stimuli. Participants were presented with new instructions informing them that they would ‘travel across different galaxies’ while still experiencing attacks. However, due to a malfunction, they would no longer be able to use the joystick to defend themselves against attacks. Their goal now was to gather information to further their mission, namely by learning which outcome was presented most frequently alongside each stimulus. The two outcomes from Phase 1 were re-used here, alongside one new outcome (dynamite) with a different aversive noise (O3). There were 68 trials in total (17 trials for each of the 4 stimuli, presented randomly), and this phase lasted 15 min in total. In each trial, one of the 4 stimuli (4.5 s) was presented to participants followed by either an outcome (aversive noise + image of attack: bomb, rocket, or dynamite) or a message saying ‘Avoided’ (1 s). Inter-trial intervals were variable (7–9 s). Two of the scenes (S + 1 and S + 2) were paired with O1 and O2 from Phase 1, while S + 3 was paired with the new O3. The fourth scene (S-) was not associated with any noise, and always displayed ‘Avoided’ when presented on screen. All S + s followed a 60–40 partial reinforcement schedule. The pairings of different outcomes with stimuli were counterbalanced across participants. After the phase concluded, participants were asked to explicitly inform the experimenter which stimuli was paired with each outcome. Participants also rated how confident they were in their stimulus-outcome pairings on a scale from 1–9. In addition, participants rated once more on a scale from 1–9 how much the space scenes appealed to them.

### Phase 3: pavlovian-to-instrumental transfer

This phase tests the ability of Pavlovian cues (space stimuli from Phase 2) to trigger instrumental avoidance responses even when they were no longer associated with any aversive outcomes (i.e., under extinction). S+ and S- images from Phase 2 were presented to participants for 30 s with an inter-trial interval of 1–3 s, At the beginning of this phase, participants were informed that their joystick had resumed working, and that they could use it to defend themselves again. However, they would no longer be notified at the start of each trial what attack was about to come. We instructed participants to continue responding with the joystick to avoid any possible attacks. No noises or images of the attacks (bomb, missile, or dynamite) were delivered throughout the phase. Participants underwent 6 blocks for each Phase 2 stimulus condition (a total of 24 trials) and for a total duration of approximately 10 min.

We ascertained here whether participants displayed significant specific (more congruent compared to incongruent responses) and general PIT (more responses towards S+ compared to S-) effects (see Statistical Analyses).

### Grip force

An isometric hand dynamometer (Biopac Systems—MP150—TSD121C—DA100C) attached to the base of the joystick was used to record grip compression (force) responses from participants. A transducer inserted inside the joystick converted grip pressure into signals fed into AcqKnowledge 3.9 (Biopac Systems). Participants were instructed to squeeze the middle of the joystick, where the transducer was located, every time they moved it left or right. Force measures obtained via grip strength over the hand dynamometer were recorded in kilograms (Kg) and extracted from the continuous signal by calculating the mean maximum amplitude per event per trial. The data were extracted per subject using AcqKnowledge 5.0 (Biopac Systems).

### Statistical analyses

Specific and general PIT effects were both quantified via percentage of responses (explicit measure of response transfer [3]) and grip force (a more implicit measure of response vigour [3]). For specific PIT, responses were classified as congruent (going in the same direction that was reinforced in the instrumental phase towards the corresponding stimulus from the Pavlovian phase) or incongruent (going in the opposite direction to what was reinforced). Mean maximum grip force corresponding to congruent and incongruent responses was also calculated. A specific PIT effect was defined as an increased proportion of, and grip force when making, congruent responses. Next, general PIT was investigated by considering the effects of S + 3 and S- on proportion of responses and grip force. The rationale for the general PIT analysis was to assess whether a similar reinforcer (i.e., the dynamite outcome, O3) *not* previously associated with a response could elicit significantly more responses than a neutral stimulus (S-). We defined greater proportion of responses and grip force towards S + 3 as being indicative of stronger general PIT. All data analyses were performed in RStudio, version 4.0.4 (R Foundation for Statistical Computing) and Jamovi 1.6.23. A statistical significance threshold of *p* < 0.05 was adopted and all tests reported here were two-tailed. The Bayes Factor (BF_10_) quantified the probability associated with the alternative hypothesis (H_1_) over the null hypothesis (H_0_). Bayes factors can be used for the evaluation of multiple hypotheses without the need to apply multiple comparisons corrections [44–46], thus we only applied multiple corrections comparisons for purely frequentist analyses (i.e., the correlations, see Results and Supplementary). Data and analysis code can be accessed on the Open Science Framework: https://osf.io/pxfhz/. See Supplementary for detailed description of statistical analysis conducted.

## Results

### Questionnaire results (Table 1)

Across all participants, OCD had greater OCI scores compared to CTL (Twj_1,50.71_ [Welch-James Test statistic] = 97.42, *p* < 0.001). Within adults, OCD had elevated trait (t_36_ = 7.52, *p* < 0.001) and state anxiety (t_36_ = 4.21, *p* < 0.001), and depression (t_36_ = 4.70, *p* < 0.001) scores. Within youths, OCD also showed increased anxiety (t_41_ = 5.91, *p* < 0.001) and depression scores (t_41_ = 7.91, *p* < 0.001).

### PIT task results (overall in Table S1)

CTL and OCD (across age groups) learnt Phase 1 instrumental (Table S2) and Phase 2 Pavlovian contingencies to the same extent (see Results in Supplementary).

There were no significant effects of Group (CTL vs OCD) over specific PIT (proportion of responses [prop. responses]: *p* = 0.32, BF_10_ = 0.36; grip force: *p* = 0.57; BF_10_ = 0.25) or general PIT (prop. responses: *p* = 0.957, BF_10_ = 0.23; grip force: *p* = 0.18; BF_10_ = 0.33), indicating that overall PIT performance was comparable between OCD and CTL (see Tables S3–S6). Statistical analysis, including means and standard deviations per performance measure per group can be found in Table S1.

### Factors modulating PIT

Given the lack of overall group differences in PIT, we sought to determine whether specific factors and learning indices differentially influenced PIT strength in OCD and CTL using Bayesian linear regression models (Tables S11–S14). We calculated indices for performance measures to enable them to be inserted as variables in the regressions (see Bayesian Regression Models section in Supplementary).

**Fig. 2:**
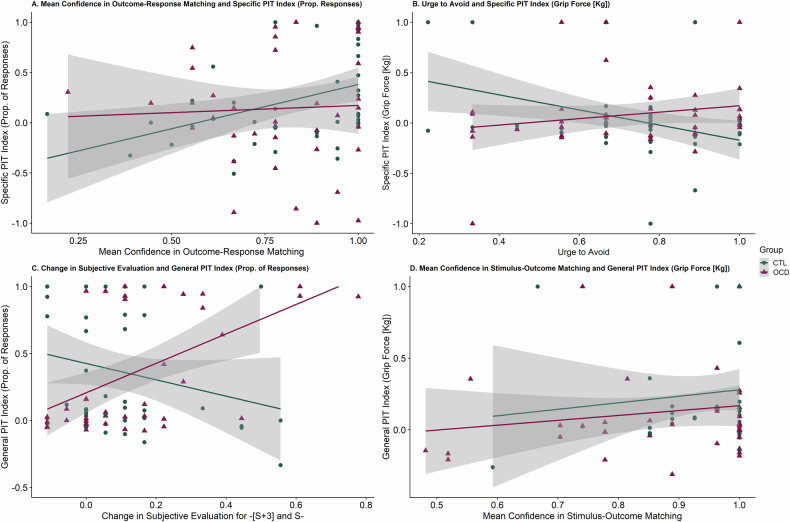
Significant Group (CTL vs OCD) interaction effects from Bayesian Regressions displayed using scatterplots. **A** Confidence in Outcome-Response Matching in OCD less associated with Specific PIT (prop. of responses) compared to CTLs. **B** Stronger positive association between avoidance urges and Specific PIT (grip force) in OCD but the opposite in CTLs. **C** Change in Subjective Evaluation for –[S + 3] and S- (greater positive change in preference for S- compared to S + 3) was associated with stronger General PIT (prop. of responses) in OCD but not in CTLs. **D** Confidence in Stimulus-Outcome Matching in OCD was less associated with General PIT (prop. of responses) compared to CTLs.

The OCD group’s specific PIT (prop. responses) was less influenced by mean confidence in Outcome-Response matching compared to CTL (Fig. 2A, Group x Mean Confidence: Estimate = −2.68, 95% CI = −3.90 to −0.79, BF_10_ = 6.05, *p* = 0.03), indicating that PIT performance increased with confidence more in CTL than OCD. Albeit, in OCD, specific PIT (grip force) was more positively influenced by urges to avoid aversive noises compared to CTL (Fig. 2B; Group x Mean Ratings of Urge to Avoid: Estimate = 1.56, 95% CI = 0.38 to 2.75, BF_10_ = 1.51, *p* = 0.011).

Next, greater positive change in preference for S- compared to S + 3 was associated with stronger general PIT (Prop. Responses) in OCD compared to CTL (Fig. 2C; Group x Mean Change in Subjective Evaluation: Estimate = 2.56, 95% CI = 0.49 to 4.61, BF_10_ = 1.86, *p* = 0.017) and in youths compared to adults (Age x Mean Change in Subjective Evaluation: Estimate = 2.61, 95% CI = 0.25 to 4.98, BF_10_ = 1.25, *p* = 0.031). Lastly, confidence ratings in Stimulus-Outcome pairings positively influenced general PIT (grip force) more strongly in CTL compared to OCD (Fig. 2D; Group x Mean Confidence: Estimate = −2.23, 95% CI = −4.30 to −0.15, BF_10_ = 1.01, *p* = 0.025).

Further analysis showed that the effects of Group x Urge to Avoid and Group x Mean Change in Subjective Evaluation (but not the interactions between Group and confidence measures) on PIT strength were driven primarily by adult participants (Adults-only – Group x Mean Ratings of Urge to Avoid: Estimate = 1.21, 95% CI = 0.06 to 2.37, BF_10_ = 2.39, *p* = 0.04; Group x Mean Change in Subjective Evaluation: Estimate = 2.37, 95% CI = 1.01 to 3.73, BF_10_ = 35.13, *p* = 0.001), while the interactions within only Youths was not significant (*p* > .05).

### Age-By-Group Interactions

**Fig. 3:**
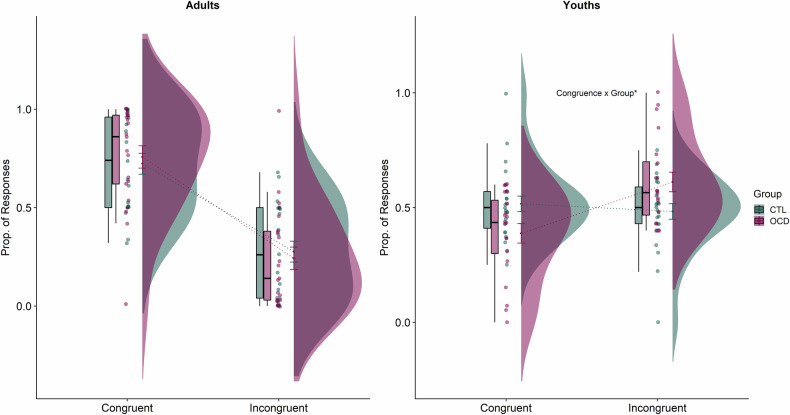
Raincloud plots (with boxplots and standard error bars) outlining Group x Age x Congruence effect for Specific PIT (prop. responses). We found a significant Group x Congruence effect in youths but not adults. Youths with OCD made more incongruent than congruent responses while youths without OCD did not differ in their proportion of congruent versus incongruent responses.

Despite a lack of overall group differences, our Bayesian analysis unexpectedly revealed moderate evidence in favour of a Group x Age interaction over specific PIT (prop. responses) (Congruence x Age x Group: F_1,81_ = 2.98, *p* = 0.088, η^2^_p_ = 0.036, BF_10_ = 3.61) – Fig. 3. Thus, we evaluated whether there were differences between OCD and CTL within each age group. A post-hoc 2×2 Mixed Bayesian ANOVA revealed that there were significant differences within youths (Group x Congruence: F_1,41_ = 5.70, *p* = 0.022, η^2^_p_ = 0.12, BF_10_ = 27.94) but not adults (*p* = 0.66, BF_10_ = 0.34). Follow-up tests indicated youth with OCD made more incongruent than congruent responses (t_19_ = −2.63, *p* = 0.016) while control youths showed no differences in proportions of congruent and incongruent responses (*p* = 0.63).

### Age Effects

**Fig. 4:**
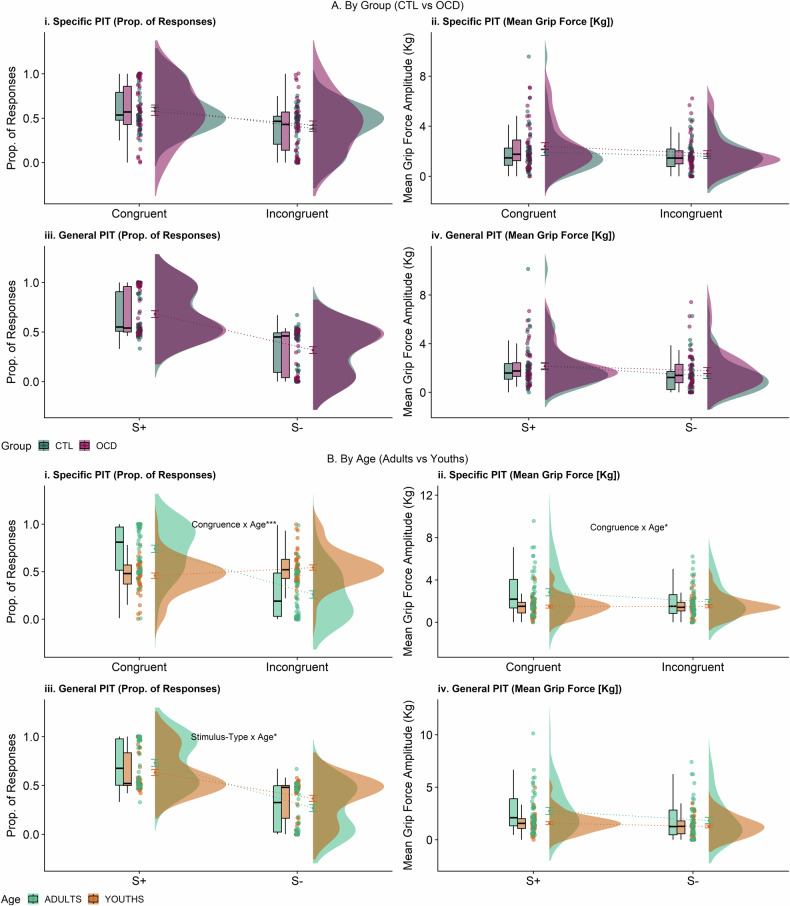
Raincloud plots (with boxplots and standard error bars) displaying main Specific and General PIT Results for Proportion (Prop.) Responses and Mean Grip Force Amplitude (in Kg). **A** Analysis by Group (OCD vs CTL) overall showed no significant effects. **B** Analysis by Age (Adults vs Youth) revealed that youths displayed significantly reduced Specific (Prop. Responses and Grip Force) and General PIT (Prop. of Responses) compared to adults. **p < 0.01, *p < 0.05.

Also unexpectedly, we uncovered significant effects of age on PIT performance (Fig. 4b). Adults displayed stronger specific and general PIT compared to youths regardless of OCD diagnosis (Specific PIT prop. responses [Age x Congruence]: F_1,81_ = 37.38, *p* < 0.001, η^2^_p_ = 0.316, BF_10_ = 2.93e + 11; Specific PIT grip force [Age x Congruence]: F_1,81_ = 5.59, *p* = 0.020; η^2^_p_ = 0.065, BF_10_ = 1.47; General PIT prop. responses [Age x Stimulus-Type]: F_1,81_ = 4.56, *p* = 0.036, η^2^_p_ = 0.053, BF_10_ = 10.41). Post-hoc tests revealed that adults made more congruent responses than incongruent responses (congruent: 0.74 ± 0.25; incongruent: 0.26 ± 0.25; t_41_ = 6.24, *p* < 0.001) and exerted greater grip strength during congruent compared to incongruent responses (congruent: 2.84 ± 2.19; incongruent: 1.92 ± 1.61; t_41_ = 2.39, *p* = 0.021) when considering specific PIT trials. Meanwhile, youths displayed no significant differentiation in proportion (Congruent: 0.46 ± 0.19; Incongruent: 0.54 ± 0.19; t_42_ = −1.53, *p* = 0.13) or grip force (Congruent: 1.48 ± 0.90; Incongruent: 1.51 ± 0.91; grip force: t_42_ = −0.28, *p* = 0.78) between congruent and incongruent responses. Next, when considering general PIT, adults (S + 3: 0.73 ± 0.23; S-: 0.27 ± 0.23; t_41_ = −6.46, *p* < 0.001) and youths (S + 3: 0.63 ± 0.19; S-: 0.37 ± 0.19; t_41_ = −4.27, *p* < 0.001) both made more responses to S + 3 than S-. However, when compared directly to youths, adults made more responses to S + 3 (t_83_ = 5.95, *p* < 0.001) and fewer responses to S- (t_83_ = −5.95, *p* < 0.001). Age did not significantly influence general PIT strength quantified by grip force (Age x Stimulus-Type: *p* = 0.077, BF_10_ = 0.45).

Incidentally, adults (specific PIT trials: 2.38 kg ± 1.46; general PIT trials: 2.29 kg ± 1.72) also applied greater force, on average (specific PIT regression: F_1,81_ = 11.18, *p* = 0.001, η^2^_p_ = 0.12, BF_10_ = 107.92; general PIT regression: F_1,81_ = 8.55, *p* = 0.004, η^2^_p_ = 0.096, BF_10_ = 57.47), when making responses compared to youths (specific PIT trials: 1.49 Kg ± 0.86; general PIT trials: 1.42 kg ± 0.81).

### Correlations

**Fig. 5:**
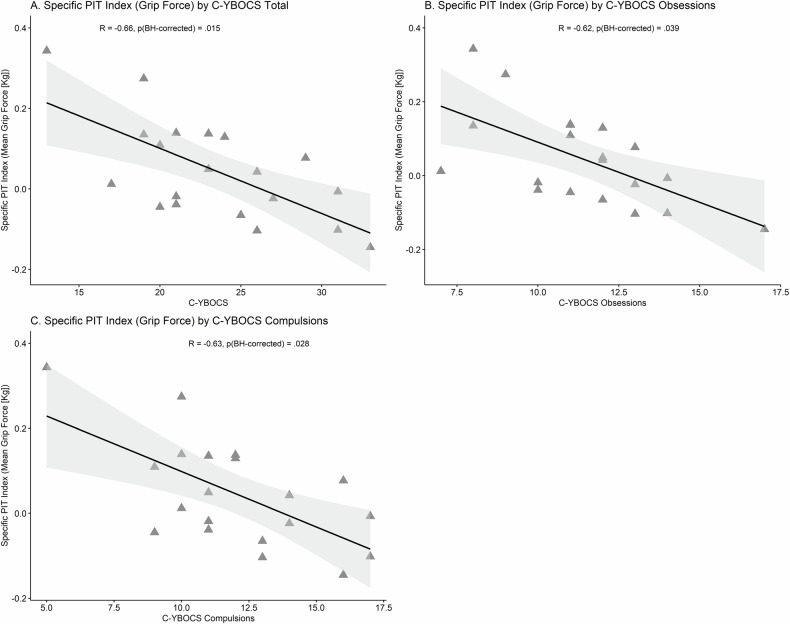
Correlations between a PIT measure and obsessive-compulsive severity. The scatterplots depict significant correlations between Specific PIT Index (Grip Force) and **A** C-YBOCS Total, **B** C-YBOCS Obsessions, and **C** C-YBOCS Compulsions. These associations were only detected in youths with OCD.

When considering all participants, there was a significant correlation between specific PIT strength (prop. of responses) and age (R = 0.42, *p*[BH-corrected] < 0.001). When considering only youth OCD participants (see Fig. 5), there was a significant negative correlation between specific PIT strength (grip force) and OCD severity measured via the CY-BOCS (R = −0.66. *p*[BH-corrected] = 0.015). Subscales of the CY-BOCS also showed significant negative correlations with the specific PIT strength (grip force) (CY-BOCS obsessions: R = −0.62, *p*[BH-corrected] = 0.039; CY-BOCS compulsions: R = −0.63, *p*[BH-corrected] = 0.028).

## Discussion

We found no evidence for changes in aversive PIT (specific or general) in OCD although there was some evidence of impaired specific PIT in the adolescent sub-group. Nonetheless, for the entire OCD sample we uncovered specific motivational factors modulating both specific and general PIT using exploratory analyses. Participants with OCD who were more motivated to avoid the aversive stimuli showed greater specific PIT, congruent with the harm avoidance model of OCD wherein compulsions are practised to prevent incoming danger, harm, and unpleasant thoughts [47–49]. This finding is evocative of prior research reporting that OCD participants display enhanced avoidance responding even under devaluation [50] and non-differentiated ventromedial prefrontal cortex hyperactivation towards safe and unsafe stimuli (operationalised as an absence of ‘safety signalling’) [15]. Additionally in our study, a stronger preference for neutral over aversive stimuli in the Pavlovian phase influenced general PIT strength in participants with OCD but not controls. Taken together, these results suggest OCD participants are relying on automatic and implicit motivational processes to drive behaviour during the PIT phase instead of goal-directed cognitive resources, consistent with a bias for model-free over model-based decision-making [51–53] and reduced goal-directed control in this population [16, 54].

By contrast, meta-cognition (confidence in explicit outcome-response and stimulus-outcome associations) in healthy controls was more informative of specific and general PIT strength. This aligns with prior research reporting that higher order cognitive processes (e.g., working memory) influence specific PIT in healthy participants [8]. Participants with OCD, by contrast, did not show this relationship between meta-cognition and PIT strength which may reflect an action-confidence dissociation previously demonstrated in OCD [22] and in the general population of people with intrusive thoughts and compulsive traits [55]. Participants with OCD often show adequate insight and meta-cognition in predictive learning tasks but do not use their knowledge to guide their actions [22, 56], reflective of clinical compulsions, which are illogical and disproportionate.

Nonetheless, the overall lack of PIT impairment in adults with OCD conflicts with recent research reporting significantly reduced specific PIT in adult patients [9] and adults with subclinical OC-traits [57]. These contradictions may be attributed to the paradigm employed and population tested; Krypotos and Engelhard (2020) recruited a non-clinical sample, while Peng et al. (2022) used a task with both appetitive and aversive components and involved participants learning to respond to rewarding stimuli and inhibit responses to punishing stimuli, being thus susceptible to elevated disinhibition in OCD patients [17, 58–61]. Our paradigm differs in that participants need to actively choose to cancel (rather than inhibit responding towards) aversive attacks, and hence our adults with OCD may have been less disadvantaged in making specific PIT responses.

Mixed findings in the PIT OCD literature reflect heterogeneous findings across other mental health disorders for PIT. Alcohol addiction studies typically report enhanced PIT effects, in line with incentive-sensitisation theory where cues previously associated with an attractive/rewarding substance are sufficient for triggering ‘wanting’ for said substance although evidence for PIT effects are less clear-cut in other substance (e.g., drugs) use disorders (see Garbusow et al. [1] and Cartoni et al. [2] for extensive reviews). Depression is reportedly associated with enhanced withdrawal in the presence of aversive PIT cues [62] but is also linked with overall reduced PIT effects for both approach and avoidance cues [63]. Next, self-rated anxiety was found to be correlated with more responding towards conditioned cues not previously associated with a reward [64] albeit a more recent study did not find any differences in PIT behaviour as a function of anxiety nor depression [65]. These divergent findings may be attributed to the highly heterogeneous PIT paradigms employed across studies (appetitive, aversive, or both) and the types of stimuli used within tasks (disorder-specific or not). It is suggested for standardisation to be applied to PIT paradigms [1] to enhance interpretation of findings aggregated across multiple studies.

Group differences in certain factors modulating PIT were driven by adult participants. In contrast to adults, youths with OCD showed impairment in specific PIT; they had a greater tendency to make incongruent responses during the PIT phase. Moreover, poor specific PIT, as measured by grip force, scaled with symptom severity in youths with OCD, further implying the disorder is impacting specific transfer ability. This suggests a developmental divergence, where adult patients show intact PIT but rely on different mechanisms from matched controls, while younger patients demonstrate less specific PIT. This finding in youths is consistent with Perkes et al.‘s (2022) findings of poor specific appetitive PIT in adolescents with OCD, associated with hypoactivity in lateral orbitofrontal cortex (OFC) and hyperactivity in medial OFC. Such neural findings may be consistent with a failure to inhibit inappropriate responses and dysfunction of the goal-directed system [54]. Overall, it does appear that both appetitive and aversive specific PIT are impaired in adolescent OCD and may provide an early behavioural marker of the disorder.

There were no noticeable differences for general PIT in youths with OCD, suggesting that they can effectively learn about general motivational properties of stimuli and apply this knowledge to their instrumental behaviour. Issues with specific PIT, instead indicate that youths with OCD are impaired at integrating multiple and distinctive Pavlovian and instrumental cues to produce behaviour although basic conditioning processes may be intact. This is compatible with reports in adolescent and paediatric OCD of poor learning and goal-directed control [31], less value-driven decision-making [21, 66] and slowness in evidence accumulation [67, 68]. These difficulties may be explained by a model of OCD [69] proposing that excessive uncertainty and an inability to synthesise prior experiences underlie dysfunctional behaviour. Instead of relying on prior evidence, patients depend on sensory feedback, which can be inaccurate, leading to enhanced compulsions to overcome such ‘not-just-right’ feelings [70].

Regardless of OCD status, PIT strength was highly developmentally sensitive in our sample, as youths showed weaker specific PIT (proportion of responses and grip force) and general PIT (proportion of responses only) compared to adults. These results cannot be attributed to poorer associative learning in youths, as learning during instrumental and Pavlovian conditioning phases was equivalent between age groups. Instead, reduced PIT suggests youths are less able integrate and generalise their learning under new contexts. Indeed, the ability to employ model-based knowledge in decision-making is a skill that only begins to emerge in adolescence [71].

Maturational differences in brain regions underlying threat perception may underlie the age-related differences in PIT susceptibility. In a prior aversive learning study, adolescents could verbally differentiate between aversive and safe stimuli, but their subjective fear ratings for each type of stimuli were less differentiated compared to adults [72]. Furthermore, adolescents recruited more subcortical regions (hippocampus and amygdala) while adults activated higher order cortical regions [dorsolateral prefrontal cortex (DLPFC)] in response to aversive versus non-aversive stimuli. Those authors inferred that the DLPFC is essential for more sensitive threat discrimination and a region that is still developing in adolescence. Youths in our study may have been similarly less sensitive to the differences between aversive and non-aversive conditioned stimuli during the PIT phase, leading to weaker transfer effects.

Recent research has elucidated that adolescents show a reduced Pavlovian bias over instrumental behaviour compared to adults and children, which translates to more exploratory behaviour, less driven by learnt contingencies [73]. One reason for this exploratory behaviour could be that adolescents are prone to overestimating the amount of uncertainty in the environment compared to adults [74]. Moreover, there is evidence that adolescents learn less effectively from punishment compared to rewards on probabilistic learning tasks [75] although they show increased punishment sensitivity on tasks with contingency reversals [76]. Greater exploration and divergent punishment learning compared with adults may underlie adolescents’ propensity for risk-taking [77] and less effective aversive PIT.

Strengths of our study include 1) recruitment of adult and youth participants, enabling greater insight into diverse developmental subtypes of the disorder, 2) our use of an aversive PIT task which is better for probing avoidance tendencies associated with the disorder compared to appetitive paradigms, and 3) inclusion of various scales (e.g., for harm avoidance and confidence) that facilitate investigation of mechanisms driving PIT. Nonetheless, we would like to emphasise that the analysis investigating mechanisms modulating PIT was exploratory in nature and results may be interpreted with caution. Future research should attempt to replicate these tentative findings with ideally larger sample sizes. Moreover, upcoming work may expand upon our study by addressing whether findings are specific to OCD, or whether they apply to other disorders of avoidance, for instance generalised anxiety disorder (although a study using a different task did not detect enhanced avoidance habits in patients with anxiety [78]). A transdiagnostic approach [79] can also be used to map PIT behavioural signatures to specific symptom dimensions. In addition, future work could involve adapting the paradigm to enable fitting of computational models to trial-by-trial PIT choice and grip data, which would better elucidate response fluctuations and latent mechanisms underlying task behaviour [9]. Lastly, we acknowledge that our exploratory age analysis may suffer from a lack of power (due to further dividing OCD and control groups by age), and that improved insight into the developmental trajectories of PIT may be obtained with larger sample sizes or, better yet, longitudinal approaches.

## Conclusions

We have identified that distinct factors contribute to the successful integration of Pavlovian and instrumental cues (measured via an aversive PIT task) in participants with OCD and healthy controls. PIT in OCD is more influenced by implicit motivational factors such as harm avoidance and subjective liking ratings, while PIT in healthy participants is associated with confidence in knowledge of learnt associations. This has implications for framing our understanding of compulsive avoidance, as being driven by automatic processes more than learnt knowledge and meta-cognition. These processes may be suitable targets for treatment with cognitive-behavioural therapy approaches. Moreover, youths with OCD show deficits in specific PIT, suggesting an impairment in evidence integration apparent only early in the disorder trajectory. Older age, regardless of OCD status, emerged as a strong predictor of specific and general PIT, suggesting that aversive PIT strength matures from adolescence to adulthood.

### Supplementary information

Supplementary Material

## Data Availability

Data and code for analyses used in this article are available online at https://osf.io/pxfhz/.
