# Supplementary material for: Compulsive avoidance in youths and adults with OCD: an aversive pavlovian-to-instrumental transfer study
Source: Transl Psychiatry. 2024 Jul 26;14:308. doi: 10.1038/s41398-024-03028-1 (PMC11282188; doi:10.1038/s41398-024-03028-1)
Supplement: Supplementary file 1 — Supplementary Material [file 41398_2024_3028_MOESM1_ESM.docx]

Statistical Analyses

Questionnaire Analysis

Two-way ANOVAS with Group and Age as independent variables were used to assess group differences in questionnaire measures. Questionnaires administered to either youths or adults only were subjected to independent-samples t-tests to assess differences between CTL and OCD groups. In the event of normality and homogeneity of variance assumption violations, the Welch-James test or Welch t-test were employed.

Main PIT Task Analysis

Learning during instrumental conditioning was assessed by calculating the percentage of correct and incorrect avoidance responses. A 2x2x2 mixed Bayesian ANOVA was conducted with Response (correct vs incorrect) as a within-subjects variable, and Group (CTL vs OCD) and Age (Adults vs Youths) as between-subjects variables.

All rating data (urge to avoid and confidence) were first converted to proportions and modelled using zero-one inflated beta Bayesian regressions with Group, Age, and Group x Age included as independent variables.

To check learning during Pavlovian conditioning, ratings of each stimulus before Pavlovian conditioning were subtracted from the ratings after Pavlovian conditioning to quantify changes in subjective evaluation per stimulus. Due to homogeneity of variance and normality violations, the Welch–James test for Robust Hypothesis Testing from the ‘welchADF’ package in RStudio^1^ was used to determine the effects of Stimulus-Type, Group, and Age over changes in subjective evaluations. A significant positive change in S- compared to the S+s would indicate learning had taken place. Post-hoc paired Wilcoxon tests with Bonferroni correction were conducted following the main Welch–James test.

Specific and general PIT effects were both quantified via percentage of responses and grip force during the PIT phase. For specific PIT, responses were classified as congruent (going in the same direction that was reinforced in the instrumental phase towards the corresponding stimulus from the Pavlovian phase) or incongruent (going in the opposite direction to what was reinforced). Mean maximum grip force corresponding to congruent and incongruent responses was also calculated. Increased proportion of responses and grip strength for congruent responses was indicative of increased specific PIT. Next, general PIT was investigated by considering the effects of S+3 and S- on proportion of responses and grip force. Greater proportion of responses and grip force would be indicative of greater general PIT. Mixed Bayesian ANOVAs were performed on percentage of responses and grip force for specific and general PIT separately. Congruence (congruent vs incongruent) was the within-subjects factor for specific PIT models while Stimulus-Type (S+ vs S-) was the within-subjects factor for general PIT models. Group and Age were inserted as between-subjects variables in all models.

For the instrumental conditioning, confidence, urge ratings, and PIT phases analyses, the Bayes Factor (BF_10_) is reported quantifying the probability associated with the alternative hypothesis (H_1_) over the null hypothesis (H_0_). Partial eta-squared (ηp^2^) was computed as an estimate of effect size for main effects and interactions. P-values for these analyses were obtained by running the equivalent frequentist test (for instance, frequentist mixed-ANOVAs) on the data.

The normality of data distributions was confirmed through low values of skewness (between -2 to +2) and kurtosis (between -7 to +7) for all variables (West, Finch, & Curran, 1995).

In the event of significant interaction effects, post hoc comparisons were conducted using pairwise t-tests with Bonferroni correction.

Factors Modulating PIT Analysis (Bayesian Regression Models)

Next, we sought to understand whether learning, confidence, subjective evaluation (liking ratings), and avoidance ratings during the Instrumental and Pavlovian Conditioning Phases informed the strength of general and specific transfer during the PIT phase^2^ and whether this differed between CTL and OCD groups.

First, for every subject, we calculated an index, separately for proportion of responses and grip force, for specific PIT by applying the formula: (congruent – incongruent)/total and an index for general transfer by applying the formula (S+3 – S-)/total. These variables were included separately as dependent variables in 4 multiple Bayesian linear regression models. We similarly calculated an index of instrumental learning from performance in the instrumental conditioning phase using the formula (correct – incorrect) / total.

The following measures were thus included as dependent variables in all of the Bayesian regressions: a) instrumental learning index, b) ‘urge to avoid outcomes’ ratings, c) mean change in subjective evaluation for S+1 and S+2 (the more negative the score, the lower the liking for S+1 and S+2 at the end of the Pavlovian phase), d) mean change in subjective evaluation for –[S+3] and S- (S+3 ratings were first multiplied by -1 to place them on the same scale as S- ratings; the more positive the overall score; the higher the preference for S- compared S+3 at the end of the Pavlovian phase) e) mean confidence ratings for explicit matching of outcomes to responses (instrumental conditioning), f) mean confidence ratings for explicit matching of stimuli to outcome (Pavlovian conditioning), g) Age (Adults vs Youths), and e) Group (CTL vs OCD). We also included interaction terms between Group, Age, and all other independent variables.

Linear models were fitted to data using the brms package^3^ in R, which uses the programming language *Stan* to implement a Markov chain Monte Carlo (MCMC) algorithm to estimate posterior distributions of parameters of interest. A weakly informative prior (a normal distribution with μ = 0 and σ = 10) was applied to estimate coefficient distributions and intercepts, while the software default for prior distributions for other model parameters (Bürkner, 2018). Four MCMC chains with random initial values and 10000 iterations (2000 warm-up) was used for sampling. We determined convergence of chains using the potential scale reduction statistic R̂. An R̂ of 1.2 was used as a cut-off for convergence^4^.

Bayes factors (BF) were calculated to determine the extent to which the alternative model (*H_1_*) had more support over the null model (*H_0_*), specifically using the Dickey Savage Density Ratio^5^. We used the following thresholds to interpret resulting BFs^6^ - BF_10_ = 1: no evidence for *H_1_, 1 <* BF_10_ < 3: anecdotal/weak evidence for *H_1_, 3 <* BF_10_ < 10: moderate evidence for *H_1_, 10 <* BF_10_ < 30: strong evidence for *H_1_, 30 <* BF_10_ < 100: very strong evidence for *H_1_,* BF_10_ > 100: extreme evidence for *H_1_.*

Correlations

We then applied Peason’s correlations between questionnaire/IQ measures and learning and performance measures from the overall PIT task (i.e., the indices calculated in the ‘Factors Modulating PIT Analysis’ section above). Benjamini-Hochberg (BH) corrections were applied to control for multiple comparisons. The BH-corrected p-values are reported.

Results

Instrumental Conditioning Phase

Instrumental learning results (Table S3) revealed a significant effect of Response-type, whereby participants overall made more correct (0.88 ± 0.14) than incorrect responses (0.12 ± 0.14) to the outcomes in the Instrumental Phase (Phase 1) (F_1,81_ = 601.46, *p* < .001, η^2^_p_ = 0.88, BF_10_ = 7.61e+74). There were no significant Group x Response (*p* = .89, BF_10_ = 0.24), Age x Response (*p* = .25, BF_10_ = 0.80), or Age x Group x Response (*p* = .43, BF_10_ = 0.54) effects, indicating the OCD and CTL as well as adults and youths learnt the outcome-response associations to the same extent.

Analysis of explicit responses revealed 32/39 OCD (Adults: 16/19 [2 missing], Youths: 16/20) and 40/43 CTL participants (Adults: 21/21, Youths: 19/22 [1 missing]) correctly matched both outcomes with the appropriate response. We verified that all those who made incorrect explicit responses towards both outcomes still showed mostly correct responses (proportion range: 0.81 – 1.0). Nonetheless, there were two youth CTL participants who achieved less than 50% correct responses in this phase despite matching the outcomes to responses correctly. For confidence ratings in Outcome-Response accuracy, there was no significant effect of Group (*p* = .44, BF_10_ = 0.063) or Age (*p* = .18, BF_10_ = 0.058).

Ratings of urge to avoid the aversive stimuli were comparable between OCD and CTL (Group: *p* = .82, BF_10_ = 0.026) and between youths and adults (Age: *p* = .24, BF_10_ = 0.081).

Pavlovian Conditioning Phase

A Welch-James test on the changes in subjective evaluation for the stimuli in the Pavlovian phase showed a significant effect of Stimulus-Type (S+1: -0.063 ± 0.15; S+2: 0.072 ± 0.16; S+3: -0.086 ± 0.16; S-: 0.072 ± 0.16; Twj_3,62.5_ = 13.18, p < .001). Post-hoc Wilcoxon tests revealed that S- showed a greater positive change in subjective evaluation compared to the S+ (comparison to S-; S+1: Z = -4.40 *p* < .001, Wilcoxon’s r = 0.41; S+2: Z = -5.03, *p* < .001, Wilcoxon’s r = 0.55; S+3: Z = -5.48, *p* < .001, Wilcoxon’s r = 0.56; other comparisons: *p* > .05); hence participants correctly learnt that S- was the non-aversive stimulus. There were no significant interaction effects of Group (*p* = 0.53) or Age (*p* = 0.58) with Stimulus-Type.

Analysis of explicit responses during this phase showed 34/39 OCD (Adults: 18/19 [2 missing due to technical issue], Youths) and 39/43 CTL (Adults: 20/21, Youths: 19/22 [1 missing due to technical issue]) accurately matched all stimuli to outcomes. For participants who incorrectly matched at least 2 stimuli to outcomes, we checked whether subjective stimuli ratings depicted learning (i.e., positive rating changes for S- and negative rating changes for S+). Three participants (1 youth CTL and 2 youth OCD) provided mostly incorrect matches and their change in ratings for S+s and S- were not indicative of learning. There were no significant effects of Group (*p* = 0.13, BF_10_ = 0.088) or Age (*p* = 0.49, BF_10_ = 0.045) on confidence ratings for Stimulus-Outcome matching.

PIT Phase

When assessing specific PIT, we found a significant effect of congruence for proportion of responses (Table S4; F_1,81_ = 16.72, p< .001, η^2^_p_= 0.17, BF_10_ = 5320.95) meaning that participants were making more congruent (0.60 ± 0.26) than incongruent responses (0.40 ± 0.26), and hence successful specific PIT was induced in participants. Grip force was a less significant indicator of specific PIT strength as shown by our Bayesian analysis (Table S5; F_1,81_ = 5.35, p = 0.023, η^2^_p_= 0.062, BF_10_ = 0.79). Next, we found evidence for successful general PIT induced in participants, as a greater proportion of responses (Table S6; S+3: 0.68 ± 0.22; S-: 0.32 ± 0.22; F_1,81_ = 57.98, p < .001, η^2^_p_= 0.42, BF_10_ = 2.10e+17) and stronger grip force (Table S7; S+3: 2.15 ± 1.70; S-: 1.55 ± 1.51; F_1,81_ = 12.47, p < .001, η^2^_p_= 0.13, BF_10_ = 2.51) was directed towards S+3 as compared to S-.

To ensure robustness of results, we repeated the PIT analysis but this time excluding the two youths who answered below 50% in the instrumental conditioning phase and the 3 youths who did not learn outcome-response associations. We confirmed that these exclusions did not alter the results (see Tables S8 – S11).

Moreover, main PIT analyses were conducted controlling for medication status; all medication effects were non-significant (*p* > .05, BF_10_<1).

Supplemental Tables

Table S1: Means and standard deviations [M(SD)] per task measure per group

|  | **CTL (N = 44)** | | **OCD (N = 41)** | | | **STATISTICS** |
| --- | --- | --- | --- | --- | --- | --- |
| **Variable** | **ADULTS (N = 21)** | **YOUTHS (N = 23)** | **ADULTS (N = 21)** | **YOUTHS (N = 20)** | |  |
| Prop. Correct during Instrumental Conditioning Phase | 0.911 (0.121) | 0.851 (0.174) | 0.882 (0.122) | 0.871 (0.142) | | Age, Group, and Group x Age: p > .05, BF_10_ ≤ 1 |
| Mean Change in Subjective Evaluation (Pavlovian Phase) | S+1: -0.0529 (0.130), S+2: -0.0847 (0.149), S+3: -0.0899 (0.174), S-: 0.0741(0.154) | S+1: -0.0145 (0.155), S+2: -0.0145 (0.155), S+3: -0.0531(0.150), S-: 0.0531 (0.164) | S+1: - 0.101(0.164), S+2: -0.111(0.183), S+3: -0.116(0.134), S-: 0.0899(0.210) | S+1: -0.0889 (0.147), S+2: - 0.0833(0.119), S+3: -0.0889(0.186), S-: 0.0722(0.104) | Age, Group, and Group x Age: p > .05 | |
| Specific PIT (Prop. Congruent Responses) * | 0.723 (0.243) | 0.517 (0.163) | 0.758 (0.261) | 0.388 (0.190) | **Age**: F_1,81_ = 37.38, *p*<.001, η^2^_p_ = 0.316, BF_10_ = 2.93e+11; **Age x Group x** **Congruence**: F_1,81_ = 2.982, p = .088, η^2^_p_= 0.036, BF_10_ = 3.61 | |
| Specific PIT (Grip Force [Kg]) * | Congruent: 2.800 (2.151)  Incongruent: 1.906 (1.390) | Congruent: 1.19 (0.695)  Incongruent: 1.326(0.909) | Congruent: 2.890 (2.271)  Incongruent: 1.930 (1.835) | Congruent: 1.900 (0.942)  Incongruent: 1.713 (0.893) | **Age**: F_1,81_ = 5.587, *p* = .020; η^2^_p_ = 0.065, BF_10_ = 1.468 | |
| General PIT (Prop. S+3 Responses)* | 0.704 (0.243) | 0.661 (0.213) | 0.760 (0.225) | 0.600 (0.193) | **Age**: F_1,81_ = 4.56, *p* = .036, BF_10_ = 10.65 | |
| General PIT (Grip Force [Kg]) | S+3: 2.930 (2.280)  S-: 1.721 (1.776) | S+3: 1.430 (1.100)  S-: 0.987 (0.756) | S+3: 2.553 (1.893)  S-: 1.958 (2.073) | S+3: 1.739 (0.677)  S-: 1.593 (0.985) | Age, Group, and Group x Age: p > .05, BF_10_ ≤ 1 | |
| Mean Confidence in Outcome-Response Matching (Instrumental Phase) | 0.915 (0.168) | 0.755 (0.221) | 0.833 (0.218) | 0.761 (0.156) | Age, Group, and Group x Age: p > .05, BF_10_ ≤ 1 | |
| Mean Confidence in Stimulus-Outcome Matching (Pavlovian Phase) | 0.958 (0.0968) | 0.936 (0.0930) | 0.901 (0.155) | 0.848 (0.158) | Age, Group, and Group x Age: p > .05, BF_10_ ≤ 1 | |
| Urge to Avoid Outcomes | 0.677 (0.230) | 0.754 (0.193) | 0.693 (0.230) | 0.772 (0.205) | Age, Group, and Group x Age: p > .05, BF_10_ ≤ 1 | |

Key: Prop., Proportion; PIT, Pavlovian-to-instrumental transfer; S, Pavlovian Stimulus; *p < .05 and/or BF_10_ > 1. Note: In the statistics column, items in bold represent variables showing significant effects.

Table S2: Results from Instrumental Conditioning ANOVA

| IV | Type of Effect | F (1,81) | p-value | η^2^_p_ | BF_10_ |
| --- | --- | --- | --- | --- | --- |
| Group | Between | 0 | 1 | 0 | 0.166 |
| Age | Between | 0 | 1 | 0 | 0.166 |
| Group x Age | Between | 0 | 0 | 0 | 0.216 |
| Response* | Within | 601.46 | < .001 | 0.881 | 7.61e+74 |
| Response x Group | Within | 0.020 | .887 | 2.52e-4 | 0.240 |
| Response x Age | Within | 1.326 | .253 | 0.016 | 0.803 |
| Response x Group x Age | Within | 0.618 | .434 | 0.008 | 0.535 |

Note: *p < .05 and/or BF_10_ > 1

Table S3: Results from Specific PIT (Proportion of Responses) ANOVA

| IV | Type of Effect | F (1,81) | p-value | η^2^_p_ | BF_10_ |
| --- | --- | --- | --- | --- | --- |
| Group | Between | 0 | 1 | 0 | 0.166 |
| Age | Between | 0 | 1 | 0 | 0.166 |
| Group x Age | Between | 0 | 1 | 0 |  |
| Congruence* | Within | 16.715 | <.001 | 0.171 | 5320.946 |
| Congruence x Group | Within | 0.999 | .321 | 0.012 | 0.359 |
| Congruence x Age* | Within | 37.378 | <.001 | 0.316 | 2.93e+11 |
| Congruence x Group x Age * | Within | 2.982 | .088 | 0.036 | 3.61 |

Note: *p < .05 and/or BF_10_ > 1

Table S4: Results from Specific PIT (Grip Force) ANOVA

| IV | Type of Effect | F (1,81) | p-value | η^2^_p_ | BF_10_ |
| --- | --- | --- | --- | --- | --- |
| Group | Between | 1.51 | .222 | 0.018 | 0.440 |
| Age* | Between | 11.18 | .001 | 0.121 | 107.92 |
| Group x Age | Between | 1.06 | .307 | 0.013 | 0.3755 |
| Congruence* | Within | 5.349 | .023 | .062 | 0.791 |
| Congruence x Group | Within | 0.327 | .569 | .004 | 0.251 |
| Congruence x Age* | Within | 5.587 | .020 | .065 | 1.468 |
| Congruence x Group x Age | Within | 0.184 | .669 | .002 | 0.283 |

Note: *p < .05 and/or BF_10_ > 1

Table S5: Results from General PIT (Proportion of Responses) ANOVA

| IV | Type of Effect | F (1,81) | p-value | η^2^_p_ | BF_10_ |
| --- | --- | --- | --- | --- | --- |
| Group | Between | 0 | 1.000 | 0 | 0.166 |
| Age | Between | 0 | 1.000 | 0 | 0.166 |
| Group x Age | Between | 0 | 1.000 | 0 | 0.219 |
| Stimulus-Type* | Within | 57.984 | < .001 | 0.417 | 2.103e+17 |
| Stimulus-Type x Group | Within | 0.000293 | .957 | 0 | 0.225 |
| Stimulus-Type x Age* | Within | 4.563 | .036 | .053 | 10.41 |
| Stimulus-Type x Group x Age | Within | 1.499 | .224 | .018 | 1.13 |

Note: *p < .05 and/or BF_10_ > 1

Table S6: Results from General PIT (Grip Force) ANOVA

| IV | Type of Effect | F (1,81) | p-value | η^2^_p_ | BF_10_ |
| --- | --- | --- | --- | --- | --- |
| Group | Between | 0.441 | .509 | 0.005 | 0.242 |
| Age | Between | 8.554 | .004 | 0.096 | 57.47 |
| Group x Age | Between | 0.814 | .370 | .010 | 0.405 |
| Stimulus-Type* | Within | 12.469 | < .001 | 0.133 | 2.507 |
| Stimulus-Type x Group | Within | 1.805 | .183 | 0.022 | 0.328 |
| Stimulus-Type x Age | Within | 3.213 | .077 | 0.038 | 0.445 |
| Stimulus-Type x Group x Age | Within | 0.218 | .642 | 0.003 | 0.482 |

Note: *p < .05 and/or BF_10_ > 1

Checking results without potential non-learners

Table S7: Results from Specific PIT (Proportion of Responses) ANOVA

| IV | Type of Effect | F (1,76) | p-value | η^2^_p_ | BF_10_ |
| --- | --- | --- | --- | --- | --- |
| Group | Between | 2.95e-15 | 1.00 | 3.89e-17 | 0.171 |
| Age | Between | 0 | 1.00 | 0 | 0.171 |
| Group x Age | Between | 8.21e-15 | 1.00 | 1.08e-16 | 0.234 |
| Congruence* | Within | 13.42 | <.001 | 0.15 | 3259.52 |
| Congruence x Group | Within | 1.135 | .29 | 0.015 | 0.353 |
| Congruence x Age* | Within | 36.0 | <.001 | 0.321 | 1.14e+11 |
| Congruence x Group x Age* | Within | 3.077 | .083 | 0.039 | 3.096 |

Note: *p < .05 and/or BF_10_ > 1

Table S8: Results from Specific PIT (Grip Force) ANOVA

| IV | Type of Effect | F (1,76) | p-value | η^2^_p_ | BF_10_ |
| --- | --- | --- | --- | --- | --- |
| Group | Between | 1.650 | .203 | 0.021 | 0.423 |
| Age* | Between | 8.648 | .004 | 0.102 | 24.51 |
| Group x Age | Between | 1.192 | .278 | 0.015 | 0.454 |
| Congruence* | Within | 5.218 | .025 | 0.064 | 1.01 |
| Congruence x Group | Within | 0.258 | .613 | 0.003 | 0.227 |
| Congruence x Age* | Within | 4.529 | .037 | 0.056 | 1.047 |
| Congruence x Group x Age | Within | 0.140 | .710 | 0.002 | 0.365 |

Note: *p < .05 and/or BF_10_ > 1

Table S9: Results from General PIT (Proportion of Responses) ANOVA

| IV | Type of Effect | F (1,76) | p-value | η^2^_p_ | BF_10_ |
| --- | --- | --- | --- | --- | --- |
| Group | Between | 0 | 1.00 | 0 | 0.171 |
| Age | Between | 0 | 1.00 | 0 | 0.171 |
| Group x Age | Between | 0 | 1.00 | 0 | 0.214 |
| Stimulus-Type* | Within | 56.06 | < .001 | 0.424 | 1.56e+17 |
| Stimulus-Type x Group | Within | 0.00066 | .980 | 8.69e-6 | 0.209 |
| Stimulus-Type x Age* | Within | 3.31 | .073 | 0.042 | 3.88 |
| Stimulus-Type x Group x Age | Within | 1.31 | .257 | 0.017 | 0.807 |

Note: *p < .05 and/or BF_10_ > 1

Table S10: Results from General PIT (Grip Force) ANOVA

| IV | Type of Effect | F (1,76) | p-value | η^2^_p_ | BF_10_ |
| --- | --- | --- | --- | --- | --- |
| Group | Between | 0.664 | .418 | 0.009 | 0.265 |
| Age | Between | 7.72 | .007 | 0.092 | 33.26 |
| Group x Age | Between | 0.300 | .300 | 0.014 | 0.440 |
| Stimulus-Type* | Within | 10.675 | .002 | 0.123 | 1.91 |
| Stimulus-Type x Group | Within | 1.258 | .266 | 0.016 | 0.309 |
| Stimulus-Type x Age | Within | 3.565 | .063 | 0.045 | 0.505 |
| Stimulus-Type x Group x Age | Within | 0.399 | .530 | 0.005 | 0.316 |

Note: *p < .05 and/or BF_10_ > 1

Table S11: Results from Specific PIT (Prop. of Responses) Bayesian Multiple Regression

| Predictors | Coefficient Estimates | CI | BF10 | p-value (from frequentist linear regression) |
| --- | --- | --- | --- | --- |
| (Intercept) | -2.15 | -4.47 – 0.15 |  | .063 |
| Group | 1.12 | -1.51 – 3.77 | 0.180 | .366 |
| Age | 1.93 | -1.66 – 5.44 | 0.316 | .283 |
| Urge to Avoid Outcomes | 0.27 | -0.51 – 1.07 | 0.0483 | .481 |
| Change in Subjective Eval. in Pavlovian Phase (S+1, S+2) | 2.54 | -0.16 – 5.26 | 0.750 | .052 |
| Change in Subjective Eval. in Pavlovian Phase (S+3, S-) | 0.44 | -1.31 – 2.16 | 0.0900 | .518 |
| Instrumental Learning Index | 0.77 | 0.06 – 1.51 | 0.350 | .033 |
| **Mean Confidence in Outcome-Response Matching (Instrumental Phase)*** | 1.93 | 0.87 – 3.00 | 13.36 | < .001 |
| Mean Confidence in Stimulus-Outcome Matching (Pavlovian Phase) | 0.13 | -1.72 – 1.97 | 0.0970 | .853 |
| Urge to Avoid Outcomes x Group | 0.86 | -0.36 – 2.07 | 0.158 | .162 |
| Change in Subjective Eval. in Pavlovian Phase (S+1, S+2) x Group | -2.68 | -5.73 – 0.36 | 0.662 | .066 |
| Change in Subjective Eval. in Pavlovian Phase (S+3, S-) x Group | 0.37 | -1.70 – 2.44 | 0.105 | .843 |
| Instrumental Learning Index x Group | 0.49 | -0.66 – 1.62 | 0.0801 | .413 |
| **Mean Confidence in Outcome-Response Matching (Instrumental Phase) x Group*** | -2.68 | -3.90 – -0.79 | 6.05 | .003  *p*(BH) = 0.012 |
| Mean Confidence in Stimulus-Outcome Matching (Pavlovian Phase) x Group | 0.37 | -2.24 – 2.07 | 0.108 | .915 |
| Urge to Avoid Stimuli x Age | -0.03 | -1.42 – 1.34 | 0.0647 | .938 |
| Change in Subjective Eval. in Pavlovian Phase (S+1, S+2) x Age | -1.96 | -5.32 – 1.43 | 0.329 | .194 |
| Change in Subjective Eval. in Pavlovian Phase (S+3, S-) x Age | -1.13 | -3.45 – 1.26 | 0.177 | .295 |
| Instrumental Learning Index x Age | -0.82 | -0.66 – 1.62 | 0.184 | .111 |
| Mean Confidence in Outcome-Response Matching (Instrumental Phase) x Age | -1.75 | -3.39 – -0.12 | 0.725 | .031 |
| Mean Confidence in Stimulus-Outcome Matching (Pavlovian Phase) x Age | -0.08 | -3.27 – 3.15 | 0.156 | .924 |
| Urge to Avoid Stimuli x Group x Age | -0.89 | -1.42 – 1.34 | 0.0906 | .359 |
| Change in Subjective Eval. in Pavlovian Phase (S+1, S+2) x Group x Age | 2.84 | -1.18 – 6.91 | 0.201 | .128 |
| Change in Subjective Eval. in Pavlovian Phase (S+3, S-) x Group x Age | 0.60 | -2.23 – 3.36 | 0.166 | .580 |
| Instrumental Learning Index x Group x Age | -0.66 | -2.16 – 0.82 | 0.0732 | .374 |
| Mean Confidence in Outcome-Response Matching (Instrumental Phase) x Group x Age | 1.20 | -1.12 – 3.51 | 0.113 | .276 |
| Mean Confidence in Stimulus-Outcome Matching (Pavlovian Phase) x Group x Age | -1.07 | -4.75 – 2.52 | 0.620 | .602 |

Key: Prop., S, Pavlovian Stimulus; Eval, Evaluation; Benjamini-Hochberg Corrected p-value. Note: *p < .05 and/or BF_10_ > 1

Table S12: Results from Specific PIT (Grip Force) Bayesian Multiple Regression

| Predictors | Coefficient Estimates | CI | BF10 | p-value (from frequentist linear regression) |
| --- | --- | --- | --- | --- |
| (Intercept) | -0.32 | -2.57 – 1.96 |  | .703 |
| Group | -0.11 | -2.72 – 2.41 | 0.129 | .979 |
| Age | 2.40 | -1.13 – 5.92 | 0.460 | .143 |
| Urge to Avoid Outcomes | -0.68 | -1.44 – 0.08 | 0.191 | .085 |
| Change in Subjective Eval. in Pavlovian Phase (S+1, S+2) | 1.14 | -1.45 – 3.76 | 0.191 | .360 |
| Change in Subjective Eval. in Pavlovian Phase (S+3, S-) | 0.18 | -1.48 – 1.87 | 0.0880 | .775 |
| Instrumental Learning Index | 0.27 | -0.44 – 0.98 | 0.0495 | .427 |
| Mean Confidence in Outcome-Response Matching (Instrumental Phase) | 0.82 | -0.22 – 1.85 | 0.178 | .112 |
| Mean Confidence in Stimulus-Outcome Matching (Pavlovian Phase) | -0.02 | -1.83 – 1.73 | 0.0903 | .936 |
| **Urge to Avoid Stimuli x Group*** | 1.56 | 0.38 – 2.75 | 1.51 | .011  *p*(BH) = .044 |
| Change in Subjective Eval. in Pavlovian Phase (S+1, S+2) x Group | -0.87 | -3.77 – 2.05 | 0.178 | .439 |
| Change in Subjective Eval. in Pavlovian Phase (S+3, S-) x Group | -0.13 | -2.17 – 1.87 | 0.102 | .834 |
| Instrumental Learning Index x Group | 0.45 | -0.67 – 1.56 | 0.077 | .434 |
| Mean Confidence in Outcome-Response Matching (Instrumental Phase) x Group | -0.96 | -2.46 – 0.54 | 0.167 | .196 |
| Mean Confidence in Stimulus-Outcome Matching (Pavlovian Phase) x Group | -0.50 | -2.59 – 1.60 | 0.121 | .572 |
| Urge to Avoid Stimuli x Age | -0.54 | -1.87 – 0.82 | 0.0913 | .368 |
| Change in Subjective Eval. in Pavlovian Phase (S+1, S+2) x Age | -1.14 | -4.35 – 2.09 | 0.208 | .439 |
| Change in Subjective Eval. in Pavlovian Phase (S+3, S-) x Age | -1.09 | -3.43 – 1.19 | 0.175 | .293 |
| Instrumental Learning Index x Age | -0.42 | -1.41 – 0.57 | 0.0495 | .393 |
| Mean Confidence in Outcome-Response Matching (Instrumental Phase) x Age | 0.27 | -1.30 – 1.83 | 0.0844 | .716 |
| Mean Confidence in Stimulus-Outcome Matching (Pavlovian Phase) x Age | -1.95 | -5.09 – 1.26 | 0.335 | .184 |
| Urge to Avoid Stimuli x Group x Age | -0.12 | -1.99 – 1.66 | 0.0906 | .948 |
| Change in Subjective Eval. in Pavlovian Phase (S+1, S+2) x Group x Age | 0.46 | -3.42 – 4.33 | 0.201 | .747 |
| Change in Subjective Eval. in Pavlovian Phase (S+3, S-) x Group x Age | 0.80 | -1.88 – 3.50 | 0.166 | .489 |
| Instrumental Learning Index x Group x Age | -0.09 | -1.54 – 1.36 | 0.0733 | .917 |
| Mean Confidence in Outcome-Response Matching (Instrumental Phase) x Group x Age | 0.09 | -2.12 – 2.31 | 0.113 | .946 |
| Mean Confidence in Stimulus-Outcome Matching (Pavlovian Phase) x Group x Age | 2.83 | -0.78 – 6.43 | 0.620 | .102 |

Key: Prop., S, Pavlovian Stimulus; Eval, Evaluation; Benjamini-Hochberg Corrected p-value. Note: *p < .05 and/or BF_10_ > 1

Table S13: Results from General PIT (Proportion of Responses) Bayesian Multiple Regression

| Predictors | Coefficient Estimates | CI | BF10 | p-value (from frequentist linear regression) |
| --- | --- | --- | --- | --- |
| (Intercept) | -1.09 | -3.39 – 1.22 |  | .364 |
| Group | 1.53 | -1.11 – 4.17 | 0.254 | .263 |
| Age | 0.53 | -3.07 – 4.18 | 0.191 | .801 |
| Urge to Avoid Outcomes | 0.59 | -0.19 – 1.36 | 0.177 | .136 |
| Change in Subjective Eval. in Pavlovian Phase (S+1, S+2) | -1.11 | -3.80 – 1.62 | 0.190 | .395 |
| **Change in Subjective Eval. in Pavlovian Phase (S+3, S-)*** | -2.09 | -3.80 – -0.34 | 1.213 | .019 |
| Instrumental Learning Index | 0.68 | -0.05 – 1.41 | 0.199 | .065 |
| Mean Confidence in Outcome-Response Matching (Instrumental Phase) | 1.11 | 0.04 – 2.17 | 0.441 | .041 |
| Mean Confidence in Stimulus-Outcome Matching (Pavlovian Phase) | -0.30 | -2.13 – 1.50 | 0.0965 | .745 |
| Urge to Avoid Stimuli x Group | -0.45 | -1.68 – 0.75 | 0.0834 | .449 |
| Change in Subjective Eval. in Pavlovian Phase (S+1, S+2) x Group | 0.28 | -2.76 – 3.31 | 0.160 | .809 |
| **Change in Subjective Eval. in Pavlovian Phase (S+3, S-) x Group*** | 2.56 | 0.49 – 4.61 | 1.857 | .017  *p*(BH) = .068 |
| Instrumental Learning Index x Group | -0.28 | -1.45 – 0.89 | 0.0649 | .621 |
| Mean Confidence in Outcome-Response Matching (Instrumental Phase) x Group | -1.61 | -3.14 – -0.04 | 0.602 | .044 |
| Mean Confidence in Stimulus-Outcome Matching (Pavlovian Phase) x Group | 0.25 | -1.90 – 2.45 | 0.114 | .825 |
| Urge to Avoid Stimuli x Age | -0.58 | -1.95 – 0.81 | 0.0992 | .423 |
| Change in Subjective Eval. in Pavlovian Phase (S+1, S+2) x Age | -0.16 | -3.50 – 3.21 | 0.169 | .132 |
| **Change in Subjective Eval. in Pavlovian Phase (S+3, S-) x Age*** | 2.61 | 0.25 – 4.98 | 1.247 | .031  *p*[BH] = .12 |
| Instrumental Learning Index x Age | -0.35 | -1.38 – 0.67 | 0.0650 | .488 |
| Mean Confidence in Outcome-Response Matching (Instrumental Phase) x Age | -1.24 | -2.85 – 0.40 | 0.253 | .132 |
| Mean Confidence in Stimulus-Outcome Matching (Pavlovian Phase) x Age | 0.98 | -2.35 – 4.25 | 0.204 | .552 |
| Urge to Avoid Stimuli x Group x Age | -0.25 | -2.16 – 1.64 | 0.100 | .785 |
| Change in Subjective Eval. in Pavlovian Phase (S+1, S+2) x Group x Age | 0.58 | -3.49 – 4.67 | 0.207 | .820 |
| Change in Subjective Eval. in Pavlovian Phase (S+3, S-) x Group x Age | -1.81 | -4.60 – 1.01 | 0.307 | .188 |
| Instrumental Learning Index x Group x Age | -0.12 | -1.64 – 1.42 | 0.0786 | .894 |
| Mean Confidence in Outcome-Response Matching (Instrumental Phase) x Group x Age | 0.68 | -1.68 – 2.92 | 0.142 | .559 |
| Mean Confidence in Stimulus-Outcome Matching (Pavlovian Phase) x Group x Age | -0.48 | -4.24 – 3.30 | 0.196 | .784 |

Key: Prop., S, Pavlovian Stimulus; Eval, Evaluation; Benjamini-Hochberg Corrected p-value. Note: *p < .05 and/or BF_10_ > 1

Table S14: Results from General PIT (Grip Force) Bayesian Multiple Regression

| Predictors | Coefficient Estimates | CI | BF10 | p-value (from frequentist linear regression) |
| --- | --- | --- | --- | --- |
| (Intercept) | -2.75 | -4.96 – -0.52 |  | .011 |
| Group* | 2.77 | 0.26 – 5.30 | 1.24 | .022 |
| Age* | 3.70 | 0.26 – 7.08 | 1.59 | .027 |
| Urge to Avoid Outcomes | 0.66 | -0.08 – 1.39 | 0.167 | .066 |
| Change in Subjective Eval. in Pavlovian Phase (S+1, S+2) | -1.15 | -3.70 – 1.44 | 0.190 | .185 |
| Change in Subjective Eval. in Pavlovian Phase (S+3, S-) | -1.50 | -3.11 – 0.12 | 0.420 | .069 |
| Instrumental Learning Index | 0.47 | -0.21 – 1.17 | 0.090 | .226 |
| Mean Confidence in Outcome-Response Matching (Instrumental Phase) | 0.62 | -0.35 – 1.62 | 0.108 | .197 |
| Mean Confidence in Stimulus-Outcome Matching (Pavlovian Phase) | 1.86 | 0.12 – 3.60 | 0.818 | .027 |
| Urge to Avoid Outcomes x Group | -0.72 | -1.86 – 0.43 | 0.130 | .194 |
| Change in Subjective Eval. in Pavlovian Phase (S+1, S+2) x Group | 0.01 | -2.88 – 2.87 | 0.143 | .974 |
| Change in Subjective Eval. in Pavlovian Phase (S+3, S-) x Group | 1.77 | -0.19 – 3.72 | 0.486 | .075 |
| Instrumental Learning Index x Group | -0.20 | -1.30 – 0.91 | 0.0579 | .704 |
| Mean Confidence in Stimulus-Response Matching (Instrumental Phase) x Group | -0.47 | -1.94 – 0.97 | 0.090 | .502 |
| **Mean Confidence in Stimulus-Outcome Matching (Pavlovian Phase) x Group*** | -2.23 | -4.30 – -0.15 | 1.01 | .025  *p*(BH) = .10 |
| Urge to Avoid Outcomes x Age | -1.00 | -2.31 – 0.27 | 0.210 | .104 |
| Change in Subjective Eval. in Pavlovian Phase (S+1, S+2) x Age | 0.85 | -2.39 – 3.94 | 0.184 | .613 |
| Change in Subjective Eval. in Pavlovian Phase (S+3, S-) x Age | 1.89 | -0.19 – 3.72 | 0.448 | .115 |
| Instrumental Learning Index x Age | -0.39 | -1.37 – 0.56 | 0.0671 | .400 |
| Mean Confidence in Outcome-Response Matching (Instrumental Phase) x Age | -0.86 | -2.38 – 0.67 | 0.139 | .276 |
| Mean Confidence in Stimulus-Outcome Matching (Pavlovian Phase) x Age | -2.30 | -5.39 – 0.84 | 0.439 | .108 |
| Urge to Avoid Outcomes x Group x Age | 0.30 | -1.48 – 2.05 | 0.0943 | .659 |
| Change in Subjective Eval. in Pavlovian Phase (S+1, S+2) x Group x Age | 0.34 | -3.42 – 4.24 | 0.193 | .878 |
| Change in Subjective Eval. in Pavlovian Phase (S+3, S-) x Group x Age | -1.66 | -4.34 – 0.94 | 0.283 | .230 |
| Instrumental Learning Index x Group x Age | 0.30 | -1.14 – 1.75 | 0.0757 | .651 |
| Mean Confidence in Outcome-Response Matching (Instrumental Phase) x Group x Age | 0.27 | -1.89 – 2.46 | 0.113 | .798 |
| Mean Confidence in Stimulus-Outcome Matching (Pavlovian Phase) x Group x Age | 3.28 | -0.24 – 6.80 | 0.855 | .051 |

Key: Prop., S, Pavlovian Stimulus; Eval, Evaluation; BH, Benjamini-Hochberg Corrected p-value; Note: *p < .05 and/or BF_10_ > 1

References

1. Villacorta, P. J. The welchADF package for robust hypothesis testing in unbalanced multivariate mixed models with heteroscedastic and non-normal data. *R J.* (2017) doi:10.32614/rj-2017-049.

2. Garofalo, S., Battaglia, S. & di Pellegrino, G. Individual differences in working memory capacity and cue-guided behavior in humans. *Sci. Rep.* **9**, 1–14 (2019).

3. Bürkner, P. C. Advanced Bayesian multilevel modeling with the R package brms. *R J.* **10**, 395–411 (2018).

4. Brooks, S. P. & Gelman, A. General MethoBrooks, S. P., & Gelman, A. (1998). General Methods for Monitoring Convergence of Iterative Simulations. Journal of Computational and Graphical Statistics, 7(4), 434–455.ds for Monitoring Convergence of Iterative Simulations. *J. Comput. Graph. Stat.* **7**, 434–455 (1998).

5. Wagenmakers, E. J., Lodewyckx, T., Kuriyal, H. & Grasman, R. Bayesian hypothesis testing for psychologists: A tutorial on the Savage-Dickey method. *Cogn. Psychol.* **60**, 158–189 (2010).

6. Andraszewicz, S. *et al.* An Introduction to Bayesian Hypothesis Testing for Management Research. *J. Manage.* **41**, 521–543 (2015).
